# The Changes in Cyanobacterial Concentration of β-Methylamino-L-Alanine during a Bloom Event

**DOI:** 10.3390/molecules27217382

**Published:** 2022-10-30

**Authors:** Siobhan J. Peters, Kenneth J. Rodgers, Simon M. Mitrovic, David P. Bishop

**Affiliations:** 1 Hyphenated Mass Spectrometry Laboratory (HyMaS), Faculty of Science, The University of Technology Sydney, Ultimo, NSW 2007, Australia; 2 School of Life Sciences, Faculty of Science, The University of Technology Sydney, Ultimo, NSW 2007, Australia

**Keywords:** cyanobacteria, algal toxins, BMAA, 2,4-DAB, cyanotoxins, Australia

## Abstract

β-N-methylamino L-alanine (BMAA) is a neurotoxin linked to high incidences of neurodegenerative disease. The toxin, along with two of its common isomers, 2,4-diaminobuytric acid (2,4-DAB) and N-(2-aminoethyl)glycine (AEG), is produced by multiple genera of cyanobacteria worldwide. Whilst there are many reports of locations and species of cyanobacteria associated with the production of BMAA during a bloom, there is a lack of information tracking changes in concentration across a single bloom event. This study aimed to measure the concentrations of BMAA and its isomers through the progression and end of a cyanobacteria bloom event using liquid chromatography-triple quadrupole-mass spectrometry. BMAA was detected in all samples analysed, with a decreasing trend observed as the bloom progressed. BMAA’s isomers were also detected in all samples, however, they did not follow the same decreasing pattern. This study highlights the potential for current sampling protocols that measure a single time point as representative of a bloom’s overall toxin content to underestimate BMAA concentration during a bloom event.

## 1. Introduction

Cyanobacteria, often referred to as blue-green algae, are phototrophic microorganisms that are widespread in aquatic and terrestrial environments [1]. While they are a natural part of many ecosystems, certain conditions, such as eutrophication, warmer temperatures [2], and decreased river flow rates [3], can increase growth and result in blooms [4]. Cyanobacterial blooms are increasing in prevalence and intensity primarily due to human activities contributing to improved growth conditions, including agricultural runoff, water extraction for irrigation, and rising global temperatures [5].

Cyanobacterial blooms can be a public health hazard because of the range and potency of the toxins that they produce [6]. These toxins, collectively known as cyanotoxins, include the neurotoxin β-N-methylamino L-alanine (BMAA). BMAA is a non-protein amino acid (NPAA) that became of interest after it was linked to the high incidence of amyotrophic lateral sclerosis and Parkinsonism-dementia complex (ALS-PDC) on the Pacific island of Guam [7]. BMAA was first isolated from cycad seeds in Guam, establishing a possible exposure route through the local diet [8]. It was later shown to be produced by symbiotic cyanobacteria in the coralloid roots of the cycads and to bioaccumulate up the food chain and make its way into the native food sources [7]. BMAA has since been found in cyanobacteria and diatoms globally [9,10] and in higher trophic levels [11,12], including foods for human consumption [13], and the brains of Alzheimer’s patients [14], with its neurotoxicity extensively reported in cell and animal models [15,16,17,18,19]. Despite this, there is still much debate on the toxicity mechanisms of BMAA, with many proposed neurotoxic properties but no clearly established mechanism for chronic toxicity [20].

Many Australian waterways are prone to developing conducive conditions for cyanobacterial growth, which can result in persistent and far-reaching blooms [21,22,23]. Regular, large-scale blooms present a health concern to surrounding communities, necessitating comprehensive monitoring of cyanobacteria and the toxins they produce to limit human exposure. Consequently, several cyanotoxins, such as hepatotoxic microcystins and neurotoxic anatoxins, are regularly monitored by government authorities globally [24]. However, this does not yet extend to BMAA or the two isomers it is often found alongside, 2,4-diaminobuytric acid (2,4-DAB) and N-(2-aminoethyl)glycine (AEG). In addition to exhibiting its own neurotoxicity, 2,4-DAB contributes to combined neurotoxicity when present alongside BMAA [17]. Studies examining the toxicity of AEG have reported conflicting results, with a recent report claiming it to be the most toxic of the three isomers [25] and others suggesting it to be the least toxic [17].

Previous studies have found BMAA and 2,4-DAB in a range of cyanobacterial bloom samples, including studies focusing on their presence in Australian blooms and cyanobacterial cultures [26,27]. However, to date, no study has assessed the concentrations of these toxic NPAAs across multiple time points during a single bloom event. Knowledge of the changes in concentration of BMAA and its isomers within a bloom over time has the potential to aid in recognising periods of increased risk of human exposure and may contribute to understanding the significance of toxin production to cyanobacteria. This study used liquid chromatography-triple quadrupole-mass spectrometry (LC-MS/MS) to measure the concentrations of BMAA and its isomers over time to track changes in toxin concentration during the progression of a bloom event.

## 2. Results

The concentrations of BMAA, 2,4-DAB, and AEG were determined for each sample using LC-MS/MS. The ‘free’ samples refer to the non-hydrolysed soluble fraction obtained following protein precipitation with 10% TCA. The second fraction was the hydrolysed precipitate produced by the addition of 10% TCA referred to as the ‘insoluble’ fraction. BMAA, 2,4-DAB, and AEG were quantified in two fractions from all four samples, over three different time points, with the final two samples being collected on the same day but selectively sampled with the ‘intact’ sample consisting of mostly alive cells, and the ‘decayed’ sample consisting of deteriorated scum.

**Figure 1:**
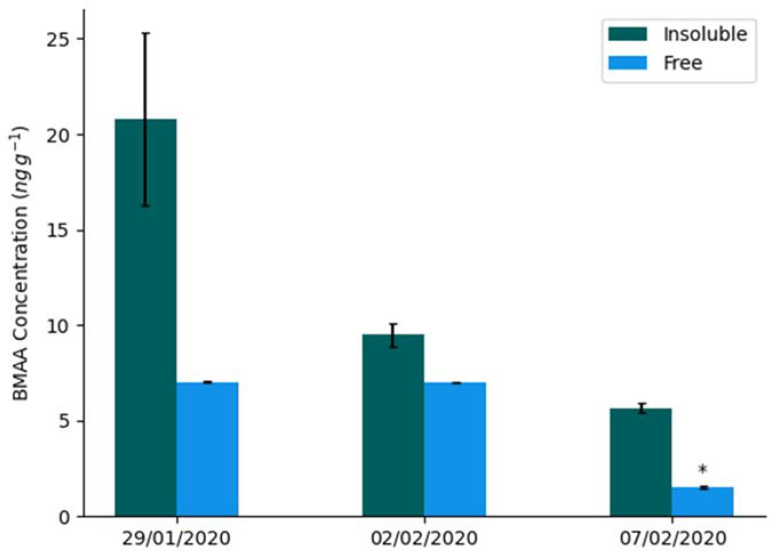
BMAA concentrations ± standard deviation in environmental cyanobacteria samples (*n* = 3, *n* = 6 for 7 February 2020 with combined decayed and the intact results). * Denotes an estimation of concentration, as this sample was detected, but below the LOQ.

**Figure 2:**
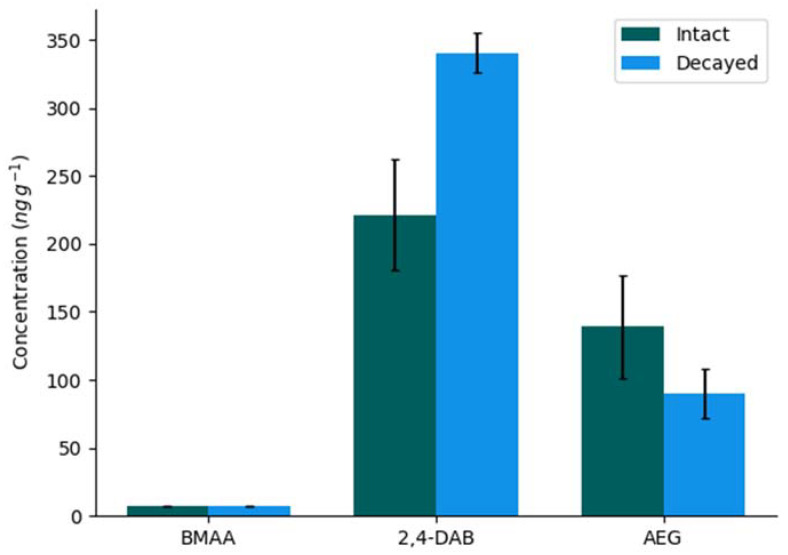
Total BMAA, 2,4-DAB and AEG concentrations ± standard deviation in samples collected on 7 February 2020 from the intact scum and the selectively sampled decaying scum (*n* = 3).

Concentrations of BMAA in both fractions decreased as time progressed (Figure 1). The insoluble protein fraction had the highest concentration of BMAA across all samples, ranging from 5.7 ± 4 ng g^−1^ dw to 21 ± 6 ng g^−1^ dw (Table 1). There was little difference between the intact and decayed sample concentrations sampled from the final time period in both fractions (Figure 2). 

2,4-DAB was only quantified in the insoluble fraction and was below the limit of quantification in the free fraction. The 2,4-DAB results from the insoluble fraction did not follow the same trend of a decreasing concentration over time as was seen in the BMAA insoluble fraction (Supplementary Materials: Figure S2), as 2,4-DAB concentrations peaked on the second sampling day before dropping down to concentrations lower than the first sampling point.

AEG was detected in both fractions from all dates, with an initial decrease and then a sharp increase in concentration in the insoluble fraction (Supplementary Figure S3). The free fraction levels were relatively consistent across all time points over the collection period (Table 1).

BMAA, 2,4-DAB, and AEG concentrations in the decayed (Table 1) intact cell samples taken from the final time point were compared (Figure 2). Whilst the BMAA concentrations showed little difference between the two samples, higher concentrations of 2,4-DAB and lower concentrations of AEG were found in the decayed sample.

## 3. Discussion

Many Australian cyanobacteria, including the bloom forming species of this study (*Dolichospermum crassum),* are known to produce BMAA and its isomers [26,27]. Some Australian cyanobacteria can also produce other toxins such as hepatotoxic microcystins, and neurotoxic saxitoxins [21] and anatoxins [28]. When assessing risk, it is essential to account for changes in cyanotoxin production during different periods of bloom development and collapse. Despite this, there has been no work to date on monitoring changing concentrations of BMAA and its isomers within a bloom event. *Dolichospermum crassum* has had both positive and negative BMAA results reported both within Australia [27] and internationally [29,30]. Considerable variation in toxin concentrations within a single study and when sampled from nearby locations have also been documented [30]. There has been little discussion about the impacts of these differences or why they occur. In the study conducted by Esterhuizen and Downing [30], 0.56 μg g^−1^ of BMAA was present in one isolated culture of *Dolichospermum*, whilst no BMAA was found in another. Similar discrepancies were also found in cultures of other genera in the same study. The variations seen in these studies have not yet been tracked over a period within the same bloom event. However, the variance in these previous studies’ results is consistent with the results of this study, in which significant differences in BMAA concentrations occurred within samples taken from the same site and bloom event across ten days. The present study’s large changes in concentration across samples taken from the same bloom but across different days highlight the problematic nature of using a single sample point as a representative result.

The previous failure to detect BMAA in some *Dolichospermum* samples [27,29,30] could result from method limitations [31], genetic factors, or conditional toxin production [32,33]. Although the role of BMAA in cyanobacteria is not fully understood, some factors that impact the production of BMAA have been explored. Environmental factors have been shown to influence BMAA production, and several studies have described how nutrient availability affects both cyanobacteria proliferation [34] and toxin production [35,36]. Nitrogen starvation has been linked to increased BMAA production in *Microcystis* sp., with nitrogen reintroduction causing a decrease in toxin levels [37]. Further, studies using the same genus of cyanobacteria have shown that high nitrogen environments correspond with stimulated cyanobacteria growth [34]. Increased growth, whilst not directly related to toxin production, is still of concern as it can facilitate the development of cyanobacterial blooms and increase exposure.

Alongside the toxin production dynamics, variances in cyanotoxin concentration for the better studied microcystins have been associated with the release of intracellular toxins into the water column or growth media upon cell death [38,39]. These studies are in contrast with the results of the current study that shows decreasing BMAA content during bloom senescence, as the microcystin concentration in the water column increased as the bloom progressed [38,39,40]. However, this is looking at the amount of toxin released, rather than the content remaining in the cyanobacteria scum. These microcystin analyses also required solid phase extraction (SPE) for clean-up and pre-concentration of the water samples. The analysis of BMAA is typically restricted to environmental cyanobacteria samples or laboratory culture isolates, limiting the capacity to determine if it also undergoes intracellular release into surrounding water.

There are currently several reports of the viability of SPE for the preconcentration and clean-up of water samples [41,42], yet relatively few have successfully detected BMAA in unspiked, natural waters [43,44]. This creates a difficulty in analysing BMAA in water samples and complicates tracking the toxin from the intra- to the extracellular and the potential release of BMAA into the water column. This, therefore, limits comparisons between the drop in concentration of BMAA over the bloom lifecycle and the increase of microcystins under similar circumstances. The release of BMAA into the water column is of notable concern as it would potentially increase routes of exposure. High concentrations in water increase the likelihood of toxin aerosolisation [45] and the uptake of BMAA by plants [46], two pathways hypothesised to result in human exposure to BMAA.

The similarity in BMAA concentrations between the decayed and the intact sample taken on the same date suggests that in this circumstance, a mechanism other than release into the water column is responsible for the decrease in concentration. If lysed cells released BMAA into the water column, there should be less in the decayed sample than in the mostly intact sample. An alternative mechanism is additionally supported by the different changes in the concentrations of the isomers. AEG changed inconsistently, with its lowest concentration being in the middle of the sampling period, whilst 2,4-DAB concentrations decreased differently to BMAA. 2,4-DAB was the only isomer to show a difference in concentration between the decayed sample than in the intact sample taken from the same date. This may be related to the presence of 2,4-DAB in high concentrations in bacterial cultures [47,48], which may be attracted to the more broken-down cyanobacteria scum from the decayed sample.

It is also important to note that the method followed in the present study included thawing frozen algal scum. This was done prior to centrifuging and the residual supernatant was discarded. Whilst retaining the pellet formed from centrifugation and discarding the supernatant is accepted general practice for BMAA extraction from cyanobacteria for both environmental samples and laboratory cultures, it could have impacts on the analysis of soluble toxins [26,30,49]. The process of freeze–thawing would result in some cell lysis [50], and it is possible that soluble NPAAs were released into the discarded supernatant. Freeze–thawing was limited to a single cycle and all samples were treated identically. Despite the potential for some loss of soluble NPAAs, this would not have influenced the insoluble fraction, in which the largest changes of BMAA concentration were observed. Additionally, changes were still observed in the free fraction, suggesting that regardless of any loss during sample preparation, the concentration of toxins across samples is still inconsistent. Future studies should aim to prepare the cyanobacteria pellet prior to freezing where possible, and analyse the supernatant using SPE for sample clean-up and preconcentration to mitigate these potential issues. Varying volumes of water are required for the current SPE methods, with one suggesting that 50 mL is the ideal volume for the best recoveries [41] and another requiring 1 L [43]. This highlights the need for considered sampling to ensure that enough sample volume is obtained to complete these analyses.

The responsive nature of the current study’s sampling regime to a bloom event and the small sample set present some limitations with this work. Limited environmental data was taken at the time of the bloom, and sampling was done inconsistently throughout, leaving an incomplete picture of the total toxicity. Between 500 mL and 1.5 L of scum sample were provided at each sampling time point, and were sub-sampled from this large volume, however these are not true technical replicates [51]. Additionally, sampling was only conducted towards the end of the bloom, with no samples provided from earlier in the growth phase. Further studies are required to establish a trend between BMAA concentration and the progression of a bloom event using technical replicates at an increased number of timepoints from the start of a bloom until cell senescence. This would allow for confirmation of the links the present study suggests, or to assess what other factors could have been affecting the changing concentrations that were seen here. Environmental monitoring and analyses of waters from the same site should also be completed alongside scum analyses. Factors such as temperature [52,53,54,55], nutrient load [53,54,56], light [53,55], and cyanobacteria growth patterns [52,54,56] are associated with varying the rates of microcystin production, and whilst many of these factors are currently unexplored regarding their impact to BMAA production, they could also play a role. These factors need to be considered in order to establish consistent and thorough monitoring. As it currently stands, BMAA sampling is typically a one-off grab sample, which, regardless of factors impacting the decline, this study has highlighted as being problematic for establishing a complete picture of toxicity.

Despite the limitations of this study, we have shown the concentration of BMAA changing throughout a cyanobacteria bloom event. This may provide some insight on the varying concentrations measured from the same genus in previous studies [27,29,30]. Our results show considerable differences in concentration over relatively short periods and suggest multiple samples need to be taken over time to properly characterise the BMAA, 2,4-DAB and AEG concentrations during bloom events.

## 4. Materials and Methods

### 4.1. Cyanobacteria Samples

Freshwater cyanobacterial samples were collected by Goulburn Valley Water (GVW) from a bloom event, with four samples taken on 3 occasions over ten days from 29 January to 7 February in the summer of 2020. Surface samples were collected from an untreated irrigation supply channel in inland Victoria, Australia. This supply channel is not used for human consumption or recreational use. The samples were collected by GVW due to the proximity to the treatment offshoot stream and were non-routine unscheduled sampling, as the bloom did not impact the extraction point. The ‘decayed’ sample was selectively sampled on the final date from parts of the bloom that had turned blue, indicating senescence and cell death. The dominant species of all samples, except the decayed sample, were identified by GVW as *Dolichospermum crassum*. The decayed sample was unable to be identified due to cellular stresses and viscosity of the sample (Supplementary Figure S1). Samples were delivered to the laboratory on ice and then stored at −20 °C until prepared for analysis.

### 4.2. Chemicals & Standards

Analytical-grade standards were used with L-BMAA hydrochloride (BMAA HCl, ≥97%) purchased from Sigma-Aldrich (Castle Hill, NSW, Australia), L-2,4-diaminobutyric acid dihydrochloride (2,4-DAB 2HCl, ≥95%) and N-(2-aminoethyl)-glycine (AEG, ≥97%) from Toronto Research Chemicals Inc. (North York, ON, Canada), and the internal standard D-2,4-diaminobutyric-2,3,3,4,4-d5 acid dihydrochloride (d5-DAB 2HCl, ≥66%) from CDN Isotopes (Pointe-Claire, QC, Canada). Sample preparation agents trichloroacetic acid (TCA, ≥99.5%), acetone (≥99.9%) and hydrochloric acid (HCl, 37%) were purchased from Sigma-Aldrich. Chrmatographic mobile phases were prepared using LC-MS grade methanol, buffered with LC-MS grade formic acid, both from Sigma-Aldrich.

### 4.3. Sample Preparation

From each time point sample, subsamples were prepared in triplicate for analysis as described by Main et al. [26] with minor modifications (described below).

#### 4.3.1. Cell Lysis

The cyanobacterial samples were thawed and centrifuged into a pellet for 20 min (Gyrozen, 1580R Centrifuge, Gimpo, Korea) at 3000× *g*. The supernatant was discarded, and the remaining pellet was freeze-dried overnight or until completely dry at 0.1 mbar and −80 °C to sublimate any remaining liquid. 20 mg of the dried pellet was transferred into a 15 mL Falcon tube, and 1 mL of 10% TCA was added to the tubes along with 20 μL of the internal standard d5-DAB (1 μg mL^−1^). Samples then underwent probe sonication (Sonics & Materials Vibra Cell, VC50T 50 W Ultrasonic Processor, Newtown, CT, USA) twice for 1.5 min at 70% power, with the tubes left on ice between repeats, to lyse the cells. Samples were then left at 4 °C overnight to allow for precipitation. Lysed cells were centrifuged at 3000× *g* for 20 min, with the subsequent supernatant transferred into a 2 mL tube. The remaining pellet was washed with 200 μL 10% TCA in water once before being transferred to a glass shell vial labelled ‘insoluble fraction’ by two washes of 200 µL 10% TCA in acetone. The subsequent supernatants from each wash were combined with the original supernatant in a tube labelled ‘free fraction.’

The free fraction was dried in a centrifugal evaporator (Thermo Fisher Scientific, Savant DNA 120 Speedvac concentrator, Waltham, MA, USA) for 24 h and then freeze-dried at 0.1 mbar to remove all liquids. This fraction was then reconstituted in 400 μL of 20 mM hydrochloric acid.

#### 4.3.2. Hydrolysis

Shell vials containing the insoluble fraction pellets were placed in a centrifugal evaporator to remove any remaining liquid. Once dried, the shell vials were placed into a vacuum hydrolysis vial with 1 mL of 6 M HCl. Oxygen was removed from the hydrolysis vial using a vacuum pump to reduce pressure to 300 mbar and then refilled with nitrogen gas. This was repeated three times to reduce oxygen levels in the vial. The hydrolysis vial was then left in an oven at 110 °C for 16 h to undergo hydrolysis with gaseous HCl. Pressure built up in the vial was released once removed from the oven, and the vials were left briefly to cool. The pellets were reconstituted in 380 µL of 20 mM hydrochloric acid and 20 µL d_5_-DAB (1 μg mL^−1^). Both fractions were then transferred to 0.2 µm membrane filter tubes (Ultrafree-MC LG Centrifugal 0.2 µL PTFE Membrane Filter) and centrifuged at 5000× *g* for 30 min. All extracts were stored at −20 °C until derivatisation.

#### 4.3.3. Derivatisation

Samples were derivatised by propyl chloroformate (PCF) using the Phenomenex^®^ EZ: Faast™ amino acid analysis kit (Phenomenex, Sydney, NSW, Australia) before analysis by LC-MS/MS. 200 μL of each fraction underwent dispersive SPE (dSPE) using EZ: Faast™ sorbent tips, derivatisation using a PCF derivatisation agent, and liquid-liquid extraction as per manufacturer’s instructions. The derivatised samples were then dried in a centrifugal evaporator before reconstitution in 50 µL of the starting mobile phase (55% methanol, 45% water).

### 4.4. Sample Analysis

The LC-MS/MS method used for the analysis of PCF-derivatised BMAA was developed and validated by Main et al. [26] and optimised on a Shimadzu Nexera UC UHPLC coupled to a Shimadzu LCMS-8060 triple quadrupole mass spectrometer. Chromatography used a Kinetex C18 column (Phenomenex; 17 µm × 2.1 × 150 mm) at a column oven temperature of 35 °C with a flow rate of 0.25 mL min^−1^. The mobile phase gradient started at 45% ultrapure water with 0.1% formic acid (solvent A) and 55% methanol with 0.1% formic acid (Solvent B). It was raised to 68% solvent B over a 10-min gradient, then increased to 100% solvent B, which was held for 5 min, before returning to starting conditions for 2 min for equilibration. Samples were prepared in triplicate, with each replicate undergoing triplicate 1 μL injections for analysis.

**Figure 3:**
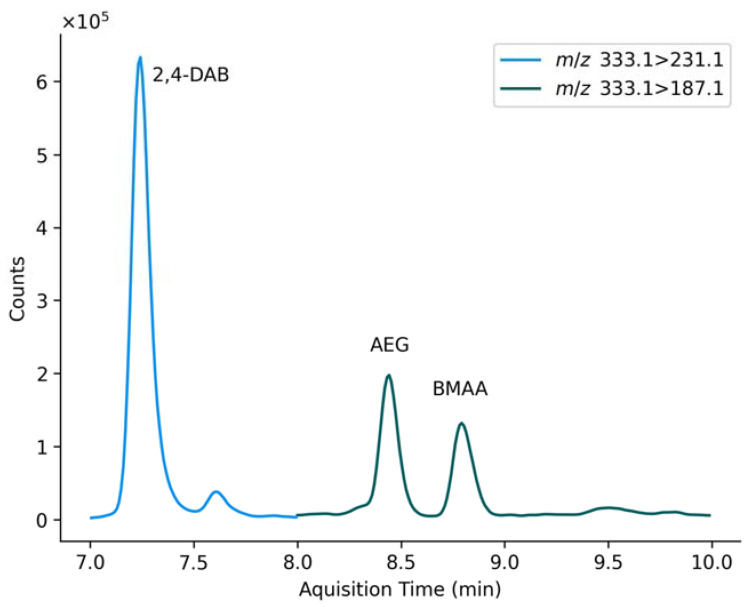
Representative chromatogram of quantitative ion transitions for 2,4-DAB (*m*/*z* 333.1 → 231.1), AEG (*m*/*z* 333.1 → 187.1) and BMAA (*m*/*z* 333.1 → 187.1) for an insoluble protein-associated sample from 29 January 2020.

An 8-point calibration curve was formed using triplicate 1 μL injections of each standard over a concentration range of 0.1–50 ng mL^−1^. The MS/MS source was set to 4 kV with a temperature of 300 °C. Nebulising and drying gas flows were set to 2 L min^−1^ and 10 L min^−1^, respectively with the heating block and desolvation line temperatures at 400 °C and 250 °C. The MS/MS was run in MRM mode for the analysis using the [M + H]^+^ transition and parameters listed in Table 2. Shimadzu LabSolutions software was used for data analysis. A representative chromatogram is shown in Figure 3.

## 5. Conclusions

The concentrations of BMAA and its isomers changed in samples collected across three time points over the period of a developed cyanobacterial bloom to the final stages of the bloom. BMAA and 2,4-DAB decreased in concentration as the bloom collapsed, while AEG increased. The concentrations of all isomers were highest in the insoluble fraction. At the final time point where intact and lysed samples were selectively collected, BMAA did not show a difference in concentration, whereas 2,4-DAB had much higher concentrations in the lysed cells. The changing concentrations of all three isomers demonstrates the need for taking multiple samples through a bloom event, and the need for improved sample preparation and analysis to measure BMAA in water samples.

## Figures and Tables

**Table 1:** Toxin concentrations ± standard deviation (*n* = 3) in environmental samples. <LOQ denotes detections below the limit of quantification.

	BMAA (ng g^−1^)		2,4-DAB (ng g^−1^)		AEG (ng g^−1^)	
Date	Free	Insoluble	Free	Insoluble	Free	Insoluble
29 January 2020	7.01 ± 0.03	20.8 ± 5.5	<LOQ	524 ± 105	5.16 ± 1.36	31.8 ± 2.7
2 February 2020	6.96 ± 0.01	9.49 ± 0.76	<LOQ	533 ± 137	4.65 ± 0.93	13.2 ± 5.6
7 February 2020 **Intact**	<LOQ	5.65 ± 0.39	<LOQ	221 ± 50	4.87 ± 0.48	134 ± 47
7 February 2020 **Decayed**	<LOQ	5.65 ± 0.22	<LOQ	340 ± 18	3.91 ± 0.98	86.0 ± 22.2

**Table 2:** MRM and retention parameters for detection of the PCF-derivatised BMAA, 2,4-DAB, and AEG. * denotes ion transition used for quantification.

Compound	Retention Time (min)	Transitions *(m/z*)		Collision Energy (eV)
2,4-DAB	7.3	333.1→	273.1 231.1 * 142.1	−20 −12 −28
d5-DAB	7.3	336.1→	276.1 * 190.1 102.1	−10 −17 −28
AEG	8.3	333.1→	187.1 * 99.1 88.1	−16 −27 −30
BMAA	8.9	333.1→	187.1 * 159.1 73.1	−16 −20 −30

## Data Availability

All data and materials support the claims made in this article.

## Supplementary Materials

The following supporting information can be downloaded at: https://www.mdpi.com/article/10.3390/molecules27217382/s1, Figure S1: Micrograph of the two samples taken from 7 February 2020; Figure S2: 2,4-DAB concentrations; Figure S3: AEG concentrations.
