# Supplementary material for: The Changes in Cyanobacterial Concentration of β-Methylamino-L-Alanine during a Bloom Event
Source: Molecules. 2022 Oct 30;27(21):7382. doi: 10.3390/molecules27217382 (PMC9658504; doi:10.3390/molecules27217382)
Supplement: Supplementary file 1 [file molecules-27-07382-s001.zip › Supplementary Information.pdf]

# Supplementary Information

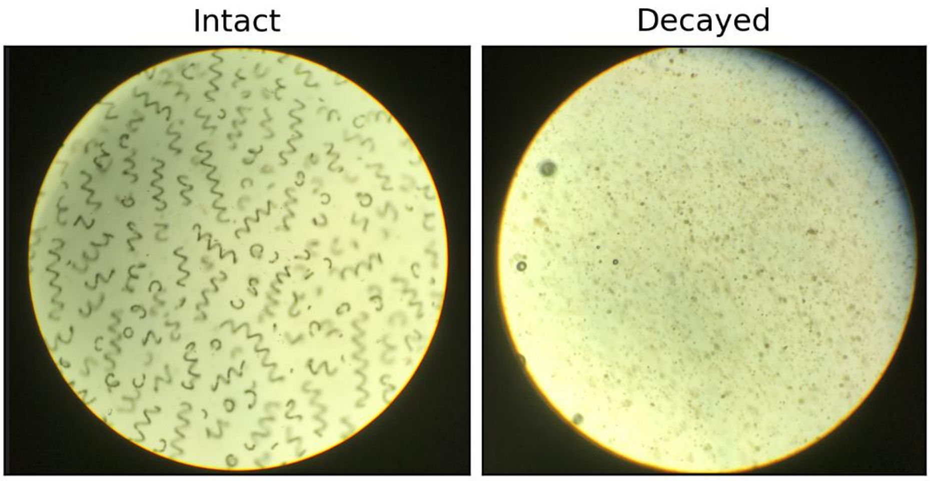


**Supplementary Figure S1.** Micrograph of the two samples taken from 07/02/2020 at 100x magnification. "Intact" is the sample taken from the bulk of the bloom, "Decayed" was selectively sampled from a part of the bloom with cell breakdown.

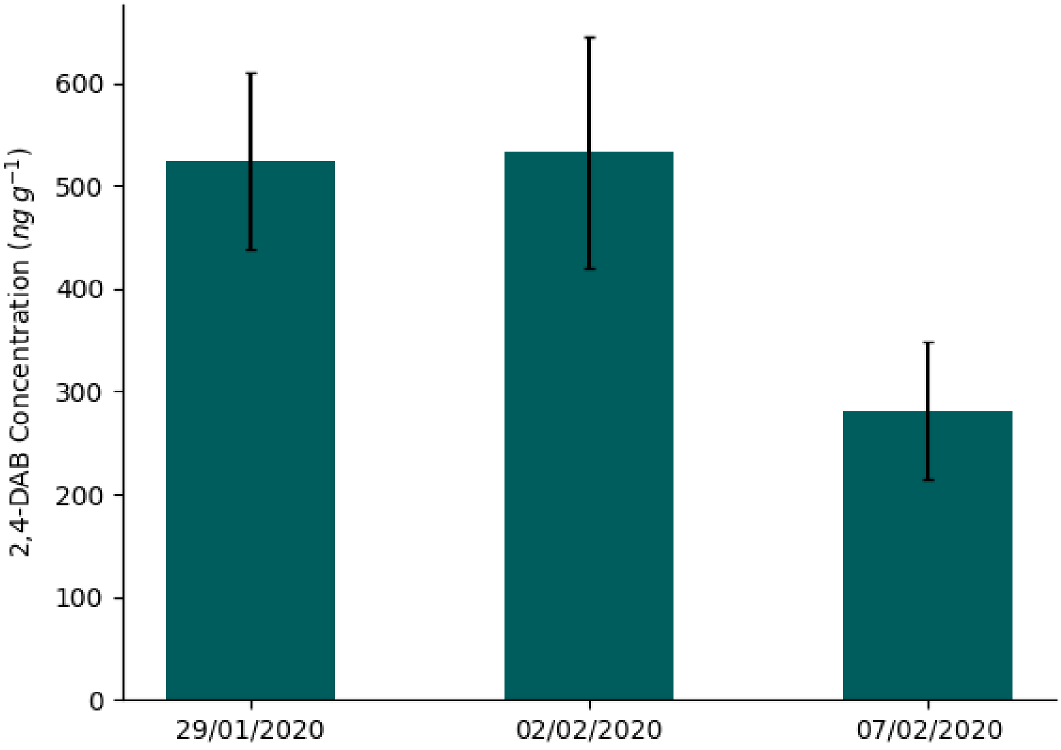


**Supplementary Figure S2** 2,4-DAB concentrations in the insoluble fraction ± standard deviation in environmental cyanobacteria samples (n=3, n=6 for 07/02/2020 with combined decayed and the intact results).

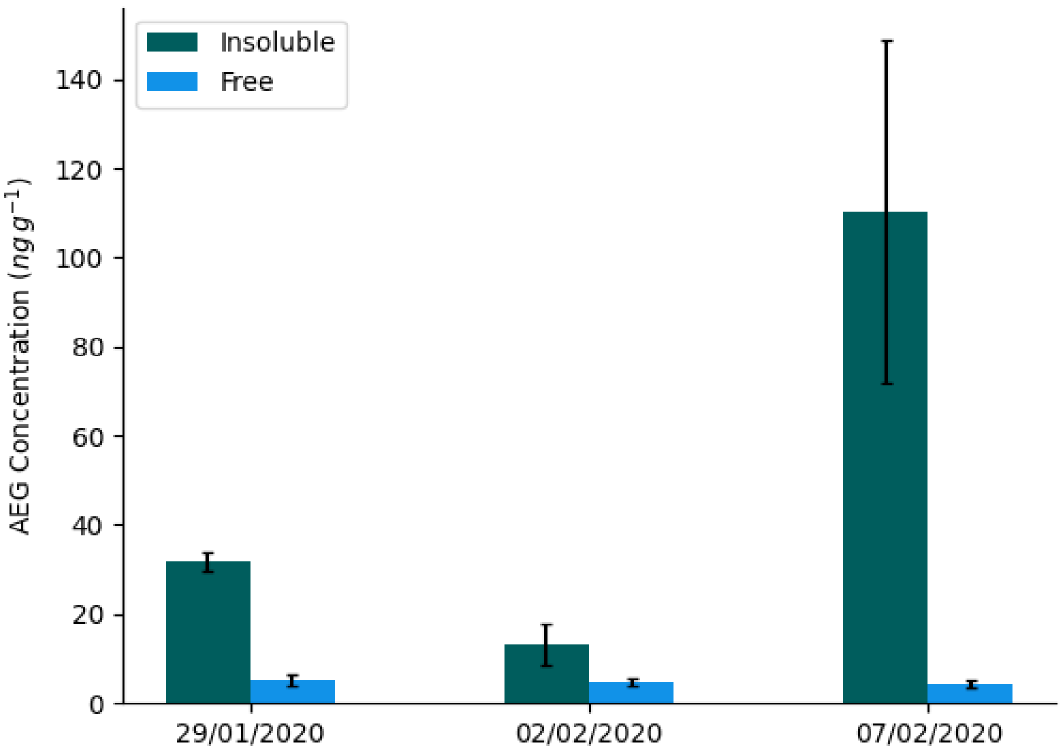


**Supplementary Figure S3** AEG concentrations ± standard deviation in environmental cyanobacteria samples (n=3, n=6 for 07/02/2020 with combined decayed and the intact results).
